# A Changing Paradigm in Heart Transplantation: An Integrative Approach for Invasive and Non-Invasive Allograft Rejection Monitoring

**DOI:** 10.3390/biom11020201

**Published:** 2021-02-01

**Authors:** Alessia Giarraputo, Ilaria Barison, Marny Fedrigo, Jacopo Burrello, Chiara Castellani, Francesco Tona, Tomaso Bottio, Gino Gerosa, Lucio Barile, Annalisa Angelini

**Affiliations:** 1 Cardiovascular Pathology and Pathological Anatomy, Department of Cardiac, Thoracic, Vascular Sciences and Public Health, University of Padua, 35128 Padua, Italy; alessia.giarraputo@phd.unipd.it (A.G.); ilaria.barison@unipd.it (I.B.); marny.fedrigo@aopd.veneto.it (M.F.); chiara.castellani@unipd.it (C.C.); 2 Laboratory for Cardiovascular Theranostics, Cardiocentro Ticino Foundation, 6900 Lugano, Switzerland; jacopo.burrello@gmail.com (J.B.); lucio.barile@cardiocentro.org (L.B.); 3 Division of Cardiac Surgery, Department of Cardiac, Thoracic, Vascular Sciences and Public Health, University of Padua, 35128 Padua, Italy; francesco.tona@unipd.it (F.T.); tomaso.bottio@unipd.it (T.B.); gino.gerosa@unipd.it (G.G.); 4 Faculty of Biomedical Sciences, Università Svizzera Italiana, 6900 Lugano, Switzerland; 5 Institute of Life Sciences, Scuola Superiore Sant’Anna, 56127 Pisa, Italy

**Keywords:** liquid biopsy, tissue biopsy, EMBs, cardiac rejection monitoring, biomarkers, heart transplant, microRNA, mRNA, gene expression profiling, exosomes

## Abstract

Cardiac allograft rejection following heart transplantation is challenging to diagnose. Tissue biopsies are the gold standard in monitoring the different types of rejection. The last decade has seen an increased emphasis on identifying non-invasive methods to improve rejection diagnosis and overcome tissue biopsy invasiveness. Liquid biopsy, as an efficient non-invasive diagnostic and prognostic oncological monitoring tool, seems to be applicable in heart transplant follow-ups. Moreover, molecular techniques applied on blood can be translated to tissue samples to provide novel perspectives on tissue and reveal new diagnostic and prognostic biomarkers. This review aims to provide a comprehensive overview of the state-of-the-art of the new methodologies in cardiac allograft rejection monitoring and investigate the future perspectives on invasive and non-invasive rejection biomarkers identification. We reviewed literature from the most used scientific databases, such as PubMed, Google Scholar, and Scopus. We extracted 192 papers and, after a selection and exclusion process, we included in the review 81 papers. The described limitations notwithstanding, this review show how molecular biology techniques and omics science could be deployed complementarily to the histopathological rejection diagnosis on tissue biopsies, thus representing an integrated approach for heart transplant patients monitoring.

## 1. Introduction

The diagnosis of acute and chronic cardiac allograft rejection remains challenging since rejection often occurs in asymptomatic patients, affecting transplanted patients’ short- and long-term outcomes [1]. Endomyocardial biopsies (EMBs) continue to be the gold standard procedure for monitoring and assessing rejection. EMBs were introduced in the cardiac transplant field about 40 years ago in many centers, first in the US and then worldwide. Monitoring EMBs for heart transplants is particularly important for post-transplanted patients, who are subjected to about 14 EMBs during the first year post-transplant [2]. This procedure provides an open window on the myocardium physiopathologic state but, like many other procedures, is prone to some limitations. First, EMBs are invasive procedures associated with some minor unavoidable clinical complications [3]; secondly, the close correlation between the clinical and histological resolution of rejection is debarred by interobserver variability and sampling errors [4]. Finally, EMBs, systemically used for surveillance during the first year after heart transplantation, represent an expensive medical procedure.

Due to these drawbacks, numerous attempts have recently been made to explore the possibility of identifying a sensitive and non-invasive approach that might be used in combination with tissue histology to reduce the frequency of biopsies [5]. Omics approaches have expanded the number of relevant biomarkers for the diagnosis of allograft rejection [6] and surveillance mainly in asymptomatic patients [7].

Liquid biopsy plays an essential role in this context with regard to the genomics and proteomics performed on blood samples, aiming to identify circulating biomarkers to achieve diagnosis and grading of rejection, bypassing the invasiveness of EMBs [8,9]. Although some of these approaches have already been introduced in several medical centers as EMB-supporting tools to monitor stable patients and reduce the number of required biopsies, the clinical implementation of these markers on a large cohort of patients is still needed. Nevertheless, EMB remains a vital source of graft status information and its potential has only partially been exploited. A specific research line has focused on identifying new molecular approaches, such as miRNomics and gene profiling, which could also be applied to EMBs as supporting technologies to improve and finely dissect histological and immunohistochemical evaluation, as well as to target pharmacological therapy [10,11].

**Figure 1:**
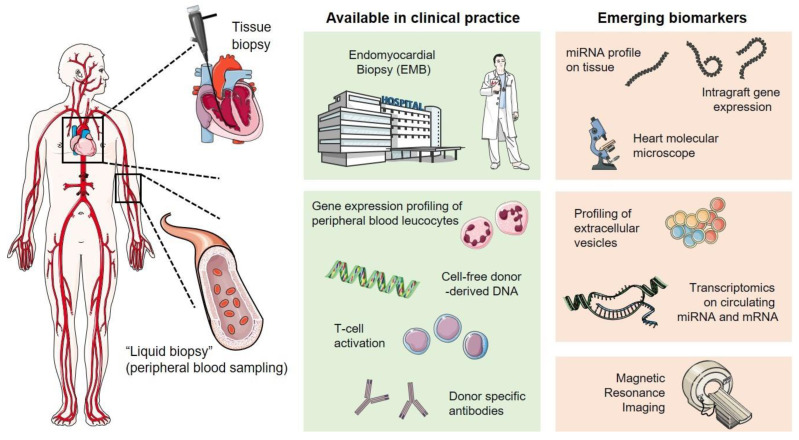
Overview of invasive and non-invasive approaches in cardiac allograft rejection monitoring.

This review covers literature from the last decade from the most used scientific databases, such as PubMed, Google Scholar, and Scopus. Starting from 192 papers, we excluded from the reviewing process all the studies that involved animal models, reviews, meta-analysis, and case studies, as well as conference abstracts/reports. We made a few exceptions however, including for some papers that represent important proofs-of-concept for novel allograft monitoring. We therefore finally included 81 papers related to clinical studies on cohorts of transplanted patients and molecular approaches performed on transplanted patients. This review presents the state-of-the-art (Figure 1) of invasive and non-invasive allograft rejection monitoring in heart transplantation.

## 2. Liquid Biopsy: Clinical Application

Initially, liquid biopsy was applied in the oncological field for diagnosis, monitoring of therapy efficacy, and assessment of progression-free survival [12,13,14]. Researchers defined it as the analysis of blood and its product to detect cellular and nuclear material derived from a tumor [15]. Thanks to its capacity to take a genetic picture of the cancer state “from a few blood drops”, it was proposed both as a companion diagnostic strategy—but also at the preclinical stage, as a population screening tool—and as a prognostic factor for outcome [15].Besides this, it showed great potential in other clinical fields, like post-transplant rejection monitoring in solid organs transplants. Good results have been achieved with these non-invasive procedures. Some methodologies, such as gene expression profiling (GEP) [8], which can detect variations in the cell transcript, have already been applied in the clinical field to monitor stable patients and reduce the number of EMBs.

### 2.1. Gene Expression Profiling of the Peripheral Blood Leucocytes

Various international multicenter studies (Cargo, Image, E-Image, and Cargo II) have investigated the role of the gene expression profiles of the peripheral blood leucocytes as genomic markers of acute rejection [7,8,9,16,17]. The first development of such an approach was an algorithm used to process a panel of 20 genes (selected starting from 252 candidates, identified through the literature and studies carried out on other transplanted organs). Eleven out of twenty genes were classifiers that could discriminate between “quiescent” vs. “moderate/severe rejection” in the validation set. Nine genes were chosen as controls. The algorithm reached an agreement of 84% in EMBs in patients with the highest grade of rejection (≥3A), measured according to the International Society for Heart and Lung Transplantation (ISHLT) classification [18]. Gene analysis converted into a score (0–40) had a negative predictive value (NPV) of 99.6% in patients demonstrating scores lower than 30 within the first year post-transplantation [8]. The Cargo II study, which included 462 patients, confirmed a GEP score < 34 to identify patients at low risk of rejection, even early after transplantation [17].

The GEP technology is commercialized as AllopMap and used in many centers in the US and Europe. Patients at >2 months, >6 months, and >1 year with a score inferior to 20, 30, and 34, respectively, were defined as low-risk patients not requiring EMBs monitoring [8,9]. This approach has been systematically compared in a clinical setting with standard routine biopsies in the IMAGE study, comprising 602 patients enrolled between six months and five years post-transplantation [7] and randomly assigned to the gene-profiling or biopsy group monitoring. Compared with the routine biopsy group, patients monitored by GEP did not experience an increased risk of severe adverse outcomes [7]. Notably, the study resulted in significantly fewer EMBs. The EImage (Early Image) study [16] evaluated the sensitivity of GEP technology during the early stage of monitoring (<6 months post-transplantation). Sixty patients were randomly assigned in a 1:1 ratio again to either the GEP or the EMB groups. The threshold for a positive GEP result was set at ≥30 for patients two to six months and ≥34 for patients six months post-transplantation. Although EImage included a small cohort of relatively low-risk patients on lower doses of corticosteroids (<20 mg), the study showed the safety and efficacy of GEP blood testing as an alternative to routine biopsies within 55 days following cardiac transplantation. There were no significant differences for primary endpoints (death, renewed transplant, hemodynamic compromise, left ventricular ejection fraction, and graft dysfunction) between patients followed-up with Allomap or EMBs [16].

Additionally, novel biomarkers can be assessed by implementing high-throughput transcriptomics profiling assays without a bias of the target selection on whole-blood samples collected during the EMB monitoring procedure. In this framework, the HEARTBiT initiative, a Canadian multicenter prospective study aimed at improving the non-invasive diagnostic performance of acute cellular rejection (ACR) events at an early stage post-transplant (during the first two months) through transcriptome profiling of nine mRNA transcripts, quantified using NanoString nCounter technology. HEARTBiT achieved a comparable diagnostic performance of non-invasive diagnostic tests currently available in clinical practice with area under the curve (AUC) values of 0.70 (95% CI, 0.57–0.83) and 0.69 (95% CI, 0.56–0.81) and with the expectation of overcoming AlloMap’s limitation to application within 55 days post-transplant [19]. A pilot clinical validation study, estimating the robustness and limitation of the previous work, assessed the potential clinical efficacy of HEARTBiT profiling. A promising linear relationship of HEARTBiT’s molecular profile with ACR diagnosis was highlighted, as well as the necessity of future longitudinal and large-scale trials [20].

The Canada-wide trial applied the GEP approach, using whole blood as starting material from patients before transplantation and from three years post-transplantation. From 1295 differentially expressed genes between subjects with acute rejection (ISHLT Grade > or = 2R) and no rejection (Grade 0R), a 12-gene biomarker panel, classifying validation samples with 83% sensitivity and 100% specificity, was identified [21]. Hollander et al. expanded and refined the 12-gene biomarker panel with a more heterogeneous cohort of cardiac transplant patients, collecting samples between one week and six months post-heart transplant [22].

Moayedi and colleagues undertook a risk evaluation of the use of GEP in monitoring acute cellular rejection and taking into account the outcomes of the AlloMap^®^ Registry in a prospective observational multicenter study [23]. They assessed short and long-term clinical outcomes in patients that received GEP for routine rejection surveillance after heart transplantation in a cohort of 1504 patients with 7969 clinic visits and records. Despite the limitation of possible selection bias in the study’s inclusion criteria, survival outcomes in contemporary heart transplant patients managed with GEP as an ACR surveillance strategy are promising [23].

Using gene-based transcriptional signaling in peripheral blood mononuclear cells in 44 patients, Mehra and Benitez reported that patients could be segregated into low, intermediate, and high risk for future rejection subsets [24]. The informative identified genes represented several biologic pathways, including T-cell activation (PDCD1), T-cell migration (ITGA4), mobilization of hematopoietic precursors (WDR40A and microRNA gene family cMIR), and steroid-responsive genes such as IL1R2, the decoy receptor for IL-2 [24].

### 2.2. Cell-Free DNA

Donor-derived cell-free DNA (dd-cf-DNA) is the DNA of donor origin in heart transplant recipients’ blood. During cellular and antibody mediated rejection, in the setting of myocyte necrosis and apoptosis, a more significant amount of dd-cf-DNA from the damaged graft is released into the blood. The detection of dd-cf-DNA is straightforward in female recipients receiving a male donor graft, undertaken by targeting the Y chromosome [25]. A molecular technique that facilitates the identification of graft-derived DNA regardless of the sex of the transplant donor or recipient is shotgun sequencing. This approach is based on sequencing cell-free circulating DNA fragments and exploiting single nucleotide polymorphisms (SNPs) genotyping information to differentiate between donor- and recipient-derived sequences. Hence, this method can identify and quantify circulating dd-cf-DNA and, during computational alignment performance, discriminate human dd-cf-DNA from microbial DNA and other erroneous material [26,27,28]. Based on this method, Snyder and colleagues introduced a new way to discriminate between donor and recipient DNA molecules, according to which increased dd-cf-DNA levels in recipients after transplantation may suggest the onset of rejection [28]. In patients experiencing acute rejection, augmented dd-cf-DNA assessed by SNPs can occur up to five months before detection on biopsy [26], indicating the potential for early diagnosis. One potential limitation using this approach is the difficulty in distinguishing graft damage due to antibody-mediated rejection (AMR) versus acute cellular rejection, implying the need for supporting follow-up tests to tune therapeutic approaches accordingly. Moreover, the complexity and cost of analyses limit its application as a clinically relevant surveillance tool. Finally, dd-cf-DNA’s targeted quantification requires genotyping of both recipient and donors, which is suitable for bone marrow and kidney transplantation but not always possible for heart transplantation. Sharon and colleagues recently addressed this issue by elaborating an algorithm that estimates dd-cf-DNA levels in the absence of a donor genotype [29]. This analysis was applied in a large, prospective, multicenter clinical trial, including patients with both ACR and AMR who received a heart transplant (HT) at least 55 days before enrolment [30]. Dd-cf-DNA testing detected acute rejection with an area under the curve of 0.64 and provided an estimated NPV of 97.1% and positive predictive value of 8.9%. The limitation of genotyping donors was also addressed by North et al., who proposed a highly sensitive and quantitative multiplexed PCR test for 94 highly informative bi-allelic SNPs [6].

North et al. developed a multiplexed allele-specific quantitative PCR method capable of the early detection of mild ACR (ISHLT 1R) in addition to higher grade ACR (ISHLT 2R and 3R), AMR, and graft vasculopathy. Using a specifically defined cut-off, the assay’s clinical performance characteristics included an NPV of 100% for grade 2R or higher ACR, with 100% sensitivity and 75.48% specificity. This test’s analytical validity facilitates conservative stratification of the probability of moderate to severe ACR as a potential companion tool for EMB in reducing the incidence of invasive biopsies, following the patients’ response to therapy [6].

A recent multicenter prospective blinded study investigated the value the ratio of cf-DNA specific to the transplanted organ, referring to the total amount of cf-DNA present in a blood sample to estimate cardiac allograft rejection. With a statistically optimized cut-off, the authors improved the dd-cf-DNA performance, reaching an NPV > 90.9% for ACR and > 99.7% for a higher grade of rejection, showing its potentiality as a novel surveillance tool thanks to its association with acute allograft rejection and a clinically applicable threshold both in adults and pediatric transplanted patients [31]. They demonstrate the feasibility of their model in detecting injury to the donor organ and as a potential clinical biomarker for AMR, elucidating new frontiers of investigation to reach statistical significance in this rejection spectrum.

Despite the recent improvement of the dd-cf-DNA technique and its advantages in non-invasive monitoring, there are still some limitations with regard to dd-cf-DNA. Concomitant kidney and liver disease, not a rare situation in heart-transplanted patients, may affect cell-free DNA clearance and may lead to an underestimation of allograft injury. Moreover, the dd-cf-DNA assay could be further limited by the long labor processing time and especially by the need for genotyping [32].

### 2.3. High-Sensitive Troponins

Cardiac troponins are well-known non-invasive tests used as reliable biological markers for several cardiovascular diseases. They have been investigated and implemented in clinical practice during the last decade. Cardiac troponins are sarcomeric structural proteins released in the bloodstream due to cardiomyocyte disruption, typically during moderate and severe ACR [33].

Myocyte damage is a mandatory pathological index of ACR for both moderate and severe events. Therefore, it has been evaluated as a potential biomarker of allograft rejection, with the aim of identifying a cutoff value for diagnosis and/or exclusion of ACR to rationalize EMB [33,34,35].

Recently, Erbel and coworkers established a high-sensitive troponin serum cutoff level of 33.55 ng/l capable of predicting death at 12 months after transplant with a sensitivity of 90.91% and a specificity of 70.97%. Besides this, survival at five years was significantly improved in patients with values below the cutoff [36]. However, contradictory results exist that show no association between allograft rejection and cardiac troponin levels [37], suggesting that high-sensitive cardiac troponin cannot be currently recommended as a tool to monitor rejection.

### 2.4. T-Cell Function

A key event in graft rejection is the activation and proliferation of the recipient’s lymphocytes, particularly T cells, which are detrimental to the long-term transplant outcome. Pharmacodynamic monitoring by direct measurement of T-cell activation and proliferation can therefore personalize immunosuppression.

The CD4 cell stimulation assay is a technology used to measure cell-mediated immunity and early response to stimulation by detecting intracellular adenosine triphosphate(iATP) synthesis in CD4+ cells selected from the blood by monoclonal antibody-coated magnetic beads. ATP can be measured by the firefly luciferase system and an assay to monitor iATP in CD4+ cells [38]. The CD4 cell stimulation assay has been approved by the US Food and Drug Administration to be applied on solid organ transplantation [38]. The assay has been used to detect iATP level released by activated CD4+ cells and their correlation with the risk of rejection or infection [38]. The significance of iATP measurement in CD4+ cells predicting acute rejection and infection is currently under investigation because different studies have found contradictory results. A published meta-analysis incorporating multiple organ transplants concluded that iATP monitoring is not suitable to identify individuals at risk of rejection or infection [39]. A drawback from the analytical standpoint is that the assay is a time-consuming, indirect cell function test requiring stimulation and a cell isolation step. Furthermore, all the studies analyzed by Ling and colleagues presented an important bias in sample selection: the numbers of rejection and infection episodes were too small to perform a robust correlation [39]. Clinical trials must generate new evidence to support the appropriate interpretation of the results.

### 2.5. Donor-Specific Antibodies

Following the consensus guidelines on the testing and clinical management issue with regard to human leukocyte antigen (HLA) and non-HLA antibodies in 2013 [40], an international consensus conference was organized in 2016 by the ISHLT to discuss current practices for detecting and quantifying circulating antibodies and validating the efficacy of therapeutic approaches [41]. Scientists agreed that equivocal results still exist with regard to the best practices for identifying antibodies of clinical relevance and their treatment. Solid-phase assays, such as the Luminex SAB assay, have been recommended to detect circulating antibodies [41]. Patients with a panel reactive antibody (PRA) <10% or donor-directed antibodies at the time of transplantation are at risk for suboptimal outcomes post-transplantation. The above-mentioned consensus conference recommended performing post-transplant monitoring for donor-specific antibodies (DSAs) at 1, 3, 6, and 12 months post-surgery [42]. For monitoring after 12 months post-surgery, the consensus supports a yearly follow-up, except for high-risk patients, who require stricter surveillance.

### 2.6. Emerging Biomarkers: Micro RNA, mRNA, Exosomes, and Microvesicles

#### 2.6.1. RNA

Various RNA molecule classes, such as protein-coding messenger RNAs (mRNA), small non-coding RNAs, long non-coding RNAs (lnc-RNAs), and other non-coding RNA molecules, have been associated with disease phenotypes, raising their potential as minimally invasive biomarkers [43,44].

lnc-RNAs are autonomously transcribed RNAs, usually longer more than 200 nucleotides, affecting significantly gene expression, e.g., with regard to chromatin regulation and T and B cells functions and differentiation. Recently, Gu and colleagues demonstrated, in a mouse model of heart transplantation, that lnc-RNAs regulate Th1 cell response during graft rejection. In this study, they compared the mRNA and lnc-RNA profiles of heart grafts and graft infiltration lymphocytes in both syngeneic and allogenic murine transplanted groups. They not only showed that A930015D03Rik and mouselincRNA1055 are highly upregulated during transplant rejection but also that they are associated with Th1 cell response through the regulation of IL12Rb1 expression. Moreover, they tested, in kidney transplanted human samples, two human lnc-RNAs expression, matching A930015D03Rik and mouselincRNA1055 separately, proving their increased expression in kidney transplant rejection samples rather than controls. This study provides a valuable proof-of-concept: lnc-RNAs can be used as innovative ACR biomarkers and potentially implemented in clinical monitoring of acute rejection episodes [44].

MicroRNA (miRNA) are small non-coding RNAs that play a role in gene expression regulation by targeting mRNA. Some miRNA are tissue- and cell-type specific and their expression level is linked directly to the pathophysiological state of organs. Extracellular circulating miRNA might be able to give us a “snapshot” of the patient’s current health state, thanks to their stability and the possibility of repeating and reproducing the measurement of their levels, which can also be undertaken using multiple frozen/thawed serum/plasma samples. For these reasons, miRNAs are suitable as non-invasive biomarkers, with several groups evaluating their diagnostic and predictor potential for allograft rejection monitoring.

In transplantation and allograft rejection monitoring, two main approaches were followed: in some studies, the authors focused on the analysis of single miRNA, while in others, they chose to analyze a group of different miRNAs that enabled them to define a characteristic pattern and pathway.

In 2013, Dewi and colleagues conducted a small pilot study to assess the potential of using serum miRNAs as acute rejection biomarkers. They investigated ten heart transplant patients, comparing serum miRNA expression levels before, during, and after rejection episodes. The analysis revealed that the levels of seven miRNAs increased during rejection. Among these, only miR-326 and miR-142-3p showed acceptable AUC values (0.86 and 0.80, respectively) (Table 1), demonstrating significant discrimination between normal and pathologic features [45]. A second study by the same authors validated the selected miRNAs in a different and more extensive cohort of patients with histologically verified acute cellular rejection (*n* = 26) and a control group of heart transplant recipients without allograft rejection (*n* = 37). The diagnostic performance in discriminating rejection vs. absence of rejection in patients using miR-142-3p and miR-101-3p revealed an AUC– ROC (Receiver Operator Characteristic) of 0.78 and 0.75, respectively [46]. Despite the more extensive and independent cohort, the numerosity was still limited, and the authors could only discriminate ACR from no-rejection status but not identify the AMR cases. However, despite this limitation, this study demonstrated that the use of circulating miRNAs in acute cardiac rejection monitoring could be beneficial [46].

Duong Van Huyen and colleagues adopted a different approach, demonstrating that miRNAs expression is regulated both on tissue and on serum. They assessed the level of 14 different miRNAs on EMBs, of which seven were differentially expressed between normal and rejecting EMB specimens. After that, the seven miRNAs were analyzed in patients’ sera, collected at identical EMB time points [47]. The analysis showed that miR-10a, miR-31, miR-92a, and miR-155 discriminated accurately between patients with and without rejection, with good yield in the external validation cohort (miR-10a AUC = 0.981, miR-31 AUC = 0.867, miR-92a AUC = 0.959, and miR-155 AUC = 0.974) [47].

Moreover, these four miRNAs facilitated the potential discriminating issue both for ACR and AMR vs. non-rejection status. However, the study was limited by the lack of an unselected prospective cohort to test miRNAs and the literature-based preselection of the miRNAs tested [47].

As showed by Van Aelst et al., miRNAs have potential as therapeutic targets for ACR. In their study, through a comparison between miRNA and mRNA expression profiles in human and mouse hearts, they identified a common signature that enabled the discrimination of rejecting and non-rejecting grafts. Hence, they demonstrated that miR-155 is overexpressed in ACR and can be a candidate target for novel therapeutics. Furthermore, they showed in a mouse model that both the knockout and the pharmacological inhibition of miR-155 delay the graft failure by reducing inflammatory infiltrate. Despite some limitations, this study highlighted the potential dual role of miRNAs not only as biomarkers but also as novel therapeutic targets [48].

#### 2.6.2. Extracellular Vesicles

Extracellular vesicles (EVs) are nanospherical membranes formed by a lipid bilayer embedded with transmembrane components, such as proteins, cholesterol, and saccharides. They envelop cytosolic proteins and nucleic acids. Based on biogenesis and size, EV classification includes exosomes, microvesicles, and apoptotic bodies. Exosomes ranging in size between 40 and 150 nm are formed and stored within subcellular compartments termed multivesicular bodies (MVBs). They are released from cells into the extracellular space upon fusion between MVBs and the cell membrane. MVBs or microparticles in the range of 100 to 1000 nm are derived from plasma membrane budding [49,50,51].

EVs act as vectors of biological information by transferring their content to target cells under basal conditions and in pathological settings. They are emerging as promising biomarker candidates for several reasons. Primarily, this is because EVs can be isolated from peripheral blood through minimally invasive sampling. Furthermore, cells that form tissues finely modulate the sorting of proteins, lipids, and nucleic acids into secretory vesicles in response to specific pathophysiological conditions [52]. Consequently, enrichment in vesicle fractions appears to provide additional diagnostic value in terms of sensitivity and specificity compared to analyses performed on unfractionated body fluids [53]. This enrichment may overcome the limitation of detecting biomarkers circulating in very low concentrations, usually below the test sensitivity routinely used in clinical practice. Our recent study analyzed the phospholipid content of vesicles, demonstrating a specific signature enabling differentiation between patients who underwent myocardial infarction and controls. However, analysis in whole unfractionated plasma resulted in the loss of the specific signature [53].

Vesicles from platelets, endothelial cells, monocytes, and leukocytes form the major component within the circulating exosomal fraction [54]. Furthermore, they are involved in immune responses, inflammation, and coagulation processes [55,56,57]. Hence, acute and chronic conditions affecting an altered inflammatory response are often associated with a systemic release of EVs containing specific proteins, nucleic acids, and/or lipids, conveying a distinct signature that is potentially relevant as a biomarker [56,57].

In this context, circulating EVs might represent a non-invasive tool for monitoring early post-transplant inflammatory responses in heart transplant recipients, supporting EMBs. We recently proposed and validated a protocol to characterize the surface antigens of circulating vesicles and assess their diagnostic performance in evaluating acute cardiac allograft rejection [58]. The application of a rapid standardized fluorescence bead-based multiplex assay combined with a supervised machine learning approach facilitated the accurate discrimination of patients with allograft rejection compared to patients without rejection. Moreover, we retrospectively confirmed the EMB-based diagnosis of the different cardiac rejection types, with a sensitivity and specificity of 100% and 85.7%, respectively. Given the high diagnostic performance, low cost, and relative usability, this method is highly promising for the characterization, monitoring, and prediction of allograft rejection, potentially reducing the number of EMBs required [58].

Kennel and colleagues performed proteomic analysis by liquid chromatography with tandem mass spectrometry on serum-derived EVs collected from cardiac transplant recipients without rejection, with ACR, and with AMR [59]. Based on relatively complex methods and instruments that analyze the entire protein content, the study demonstrated the predictive and prognostic value of EVs as biomarkers.

The significance of EVs as predictive and diagnostic biomarkers of rejection is even more promising because, besides their role as measurable indicators of distinct biological conditions [60], EVs hold functional biological characteristics in immune response modulation, thus providing consistency as tools to monitor the allograft status and improve post-transplant outcomes [60]. EVs display Major Histocompatibility Complex (MHC) class I and II, as well as adhesion and costimulatory molecules, resembling the role of antigen-presenting vesicles for allospecific T-cell activation [61]. In a murine heart transplant model, exosomes from mature dendritic cells (DCs) mediated their endocrine signaling by migrating to the regional lymph nodes. This process facilitated acute rejection by activating T cells, thus amplifying the effect of the limited number of donor DCs present in the transplanted organ [62]. In a mechanism implying contact-dependent signaling, MHC peptides carried on the EV surface derived from mature DCs efficiently primed T cells [63], playing a role in inducing tolerance in a fully MHC-mismatched rat cardiac allograft model [64]. A further indirect mechanism has been described for follicular DCs unable to synthesize MHC class II proteins. These cells passively acquire the complex by capturing circulating MHC class II-expressing EVs, presenting them to T cells as a vesicle-composed wreath [65]. In this case, DCs enhanced the stimulatory capacity of EV and the presence of cell-to-EV binding is critical to stimulate specific T cells efficiently [61,62,63,64,65,66]. Following these findings, which suggest that donor vesicles may play a role as exclusive sources of donor MHC for T-cell activation, Habertheuer et al. recently showed that transplanted hearts release donor-specific EV. In a murine model of a heterotopic heart transplant, the cardiac allograft released a distinct pool of donor MHC-specific EVs into the recipient circulation. The signal peaked during early acute rejection with high accuracy [67], enabling the developing of a highly specific and sensitive biomarker platform for allograft monitoring [67,68].

Finally, Sharma et al. recognized cardiac myosin and vimentin as tissue-restricted self-antigens that are detectable on the surface of circulating EVs and are associated with primary graft dysfunction [69]. Hence, the above-cited mechanisms are consistent because the transplanted heart is a vascularized organ at the time of placement and donor vesicles released may leak through the vascular endothelium and be trafficked into the hosts’ bloodstream.

## 3. Tissue Biopsy for Molecular Tests

The liquid biopsy is a very attractive non-invasive source of information for monitoring post-heart transplanted patients. However, the opportunity offered by the utilization of tissue biopsy for molecular tests remains pivotal. Different groups have tried to identify new biomarkers in EMBs to improve the diagnosis of rejection. EMB is the gold standard in monitoring cardiac rejection. Generally, the pathologists assess the inflammatory infiltrate and the myocardial injury through histological and immunohistochemical evaluation. This approach makes it possible to define and grade the rejection but also to modify the pharmacological therapy. Thus, EMB is an important source of information about a graft’s status, but its potential has only partially been exploited. The opportunity to investigate the rejection mechanisms directly on the tissue offers new insights into the cellular interactions and graft injury evolution over time. Different groups have tried to apply new technologies to understand the molecular pathways involved in rejection, assess the cardiac allograft status, and define new biomarkers to ameliorate the rejection diagnosis on biopsy tissue.

### 3.1. MicroRNA on Tissue

As discussed above, miRNAs are non-coding regulative molecules in numerous signaling pathways also involved in pathophysiological disorders. The investigation of miRNA expression on cardiac tissue could help in the characterization of rejection events [70].

Recently, we defined an miRNA signature to discriminate ACR, AMR, and mixed rejection (MR) on formalin-fixed and paraffin-embedded (FFPE) EMB specimens through next-generation sequencing (NGS) and reverse transcription quantitative polymerase chain reaction (RT-qPCR). Using logistic regression analysis, we created unique miRNA signatures as predictive models of each type of rejection. More than 2257 mature miRNAs were obtained from all EMBs. Each of the three rejection types showed a different miRNA profile. The logistic regression model formed by miRNAs 208a, 126-5p, and 135a-5p identified MR, whereas ACR was identified by the miRNAs 27b-3p, 29b-3p, and 199a-3p. In contrast, AMR was identified by the miRNAs 208a, 29b-3p, 135a-5p, and 144-3p [10].

Another interesting work by Nováková and colleagues aimed to identify miRNAs dysregulated on FFPE EMB specimens during ACR [11]. The authors used a stepwise backward regression method and three principal component analyses (PCA) to create an ACR SCORE model. This model uses the levels of 11 miRNAs (miR-144, -589, -146, -182, -3135b, -3605, -10, -31, -17, -1273, -4506), detected in EMBs through RT-qPCR, to assign an ACR SCORE to the specimens. If the score is above the defined cut-off value, the authors defined ACR as present with a specificity of 91% and a sensitivity of 68% [11].

Furthermore, the RT-qPCR validation of the 11 miRNAs, previously identified in EMBs through NGS, confirmed that miR-144, miR-589, and miR-182 are statistically significantly altered during rejection [11].

As stressed by Nováková et al., a remarkable limitation of studies focused on the use of microRNA as biomarkers is the lack of uniformity (Table 1). Many studies demonstrated that miRNAs are eligible biomarkers of disease; however, they were not unanimous in identifying a unique miRNA or miRNA signature applicable in clinical practice. Various methodological factors could be the source of this heterogeneity, e.g., the different protocols for RNA isolation and analysis or the nature of the EMB specimens, which incorporate many different cell types (cardiomyocytes, lymphocytes, fibroblast and endothelial cells) with different cell-specific levels of miRNA expression. Furthermore, patients’ comorbidities and personalized immunosuppressive therapy impact the cohort variability included in multiple studies, leading to a lack of consistency [11].

### 3.2. Molecular Microscope

The molecular microscope, first developed for kidney transplantation (KT), uses rejection-associated transcriptome (RAT) profiling of known kidney biopsies as a reference to generate an automated, objective, and quantitative report to offset intercenter variation [71]. Halloran and colleagues proposed this as a new method to also assess rejection in EMBs. A direct comparison of this system with EMBs guided the development of a molecular microscope heart diagnostic system called the Heart Molecular Microscope Diagnostic System (MMDx-Heart), a microarray-based technology used to evaluate the molecular status of tissue [72]. This approach relies on the hypothesis that the inflammatory lesions and molecular alterations of heart allograft rejection act similarly to KT, closely correlating histologic features of EMBs and KT biopsies [72].

An unsupervised PCA was performed based on RAT expression scores in the 1208 indication kidney biopsies and the 331 EMBs and three archetype scores were assigned in EMBs corresponding to histologic T cell-mediated rejection (TCMR), antibody-mediated rejection (ABMR), and no rejection (NR) groups. Combining the best performing probes, they achieved AUC–ROC values of 0.78, 0.65, and 0.81 for NR, TCMR, and ABMR archetypes, respectively [72]. These results showed that this system has a lower sensibility for EMBs than for kidney biopsies and a higher disagreement level between molecular and histopathologic assessments. According to the authors, this last point reflects the higher interpathological disagreement for EMB assessment highlighted by the Cargo study, particularly for cellular-mediated rejection [72]. Moreover, the authors suggested that the molecular discrepancy observed in TCMR diagnosis could be due to the Quilty effect’s presence and that the further investigation of its molecular similarities with TCMR could be very enlightening. Overall, this study demonstrated that molecular tissue analysis reflects the complexity of EMB assessment, with further investigations required to overcome the present limitations [72].

### 3.3. MRI for Non-Invasive Monitoring in Tissue

At the tissue level, graft rejection presents infiltrative inflammatory cells with an expansion of the extracellular space and necrosis. These morphostructural changes have been investigated in several studies with a cardiovascular magnetic resonance (CMR) methodology, enabling non-invasive imaging with qualitative and quantitative tissue characterization. CMR can evaluate histopathologic changes due to rejection, associated with distinct myocardial T1 and T2 relaxation times [73]. Using a multiparametric sequential approach, by combining basal T2 mapping with the basal extracellular volume fraction, improved diagnostic accuracy for transplant rejection can be achieved [74]. Therefore, a multisequential CMR examination could operate as a non-invasive tool for excluding subclinical ACR in heart transplant patients [75].

### 3.4. Gene Expression Profile in the Tissue

The value of myocardial GEP for diagnosing and identifying the predictive biomarkers of ACR was evaluated in the GET study by Bodez and Damy in 36 EMBs in 30 patients [72]. They demonstrated that the cardiac gene expression profiles of EMBs only partly matched the histological grading system, suggesting that cardiac GEP may provide earlier and more sensitive performances in diagnosing ACR and can be used as an early screening test for ACR [76].

GEP for the identification and classification of antibody-mediated heart rejection was evaluated by Loupy et al. in 110 patients [77]. The authors applied a combination of multidimensional molecular assessments to extensive phenotyping allograft biopsies to characterize anti-HLA antibodies and cellular models, demonstrating that antibody-mediated rejection in heart transplantation is driven mainly by the natural killer burden [77].

Using the NanoString nCounter technology, the expression of a set of 34 literature-derived genes reflecting the molecular correlates of antibody-mediated injury in 106 FFPE EMBs for a training cohort and 57 EMBs for a validating cohort was evaluated in a recent study by the Edmonton group [78]. The gene set selected by the authors for AMR profiling revealed a good diagnostic accuracy (approximately 70%) in identifying AMR positive cases vs. negative ones, with a sound correlation with other diagnostic methods routinely applied, such as histopathology, anti-HLA DSA, and C4d immunohistochemistry. However, the gene set was unable to differentiate pathological AMR1(I+) from ACR and normal controls, raising the question of the real value of this histopathological grade, which, according to their results, appears more similar to ACR than to AMR [79]. This very promising study highlights the need for a larger cohort of patients but also more importantly for the identification of a new set of genes capable of differentiating between AMR and other non-immunologic endothelial injuries [78].

Intragraft gene expression studies can highlight the functional status of the transplanted organ. Arteriolar vasculitis in EMBs may be pivotal in identifying high-risk episodes in transplant recipients. Gene expression analysis could help in understanding alterations in genes profile associated with vasculitis, affecting the heart allograft’s survival in long-term transplantation. From this perspective, Lin-Wang et al. conducted a retrospective study of 300 FFPE EMBs of 63 patients to determine the incidence of vasculitis and its association with ACR, AMR, and cardiac allograft vasculopathy. This study performed a gene expression analysis of the chosen transcript involved in inflammation and vascular function as evaluated by q-PCR. Their results showed that vasculitis carried worse prognostic outcomes [80].

## 4. Conclusions

Allograft rejection is a life-threatening complication of organ transplantation. Its monitoring is a fundamental step in the post-transplant follow-up.

EMBs remain the gold standard for cardiac allograft monitoring; however, the perceived need to complement the histological examination of the tissue with molecular approaches has led to the development of several molecular approaches to implementing diagnosis.

Indeed, the histological evaluation of cardiac tissue assesses the allograft’s inflammatory status; nevertheless, EMBs represent such valuable in vivo tissue that much more information needs to be retrieved from them than just the information about the inflammatory status.

Omics may improve comprehension of the allograft’s pathophysiological feature and provide pathologists and clinicians with new insights related to the graft that help in developing a personalized therapeutic approach for better management of transplanted patients. The application of several molecular techniques on cardiac tissue represented a starting point for a new era of allograft rejection diagnosis. As discussed above and described in the evaluated research (Table 1), many studies demonstrated that comprehension of the rejection process is still limited. Research must go on to dissect it. Like any invasive procedure, EMBs can cause some complications and counterbalancing these negative aspects has been researchers’ primary intent for years. Many non-invasive approaches have been designed and tested in clinical trials in the hope of identifying an alternative diagnostic tool for rejection monitoring. Several cutting-edge methods that rely on liquid biopsies have implemented procedures and clinical applications that provide the most comprehensive patient heart transplant snapshots. Recent efforts in the transplant research field have also applied these novel methods to the tissue to combine different information from various resources.

Approaches for defining the specific signature of circulating EVs from liquid biopsies show promising results but require further studies to validate their robustness and reliability through large-scale trials introduced in clinical practice.

Despite their contributions to highlighting novel and not-yet-investigated points of view in heart allograft rejection monitoring, the studies cited in this review share several common limitations: the relatively limited size of the patient cohorts and the lack of convergence towards a standard molecular profile of cardiac rejection. The GEP, CARGO, and IMAGE projects have overcome these issues. Their genetic approach can be proposed as a companion tool in diagnosis to reduce the number of EMBs performed. These kinds of processes often leverage sophisticated technologies not yet available for a large segment of transplanted patients.

Hence, the potential of genomics and transcriptomics is well-recognized and the identification of a transcriptome profile without a bias of selection could help in the definition of a shared panel of biomarkers for both the recognition of rejection and the discrimination between ACR, pathological AMR, MR, infections, and other injuries [18,79].

It is therefore crucial to recognize the need for wide-ranging studies, clinical exploiting the potential of big data analysis and machine learning techniques. As seen in the prediction system for kidney allograft loss in the iBox study [81], there is a raised awareness with regard to the personalization of follow-up procedures and therapies for future heart transplanted patients.

The improvement of novel molecular approaches in tissue and liquid biopsies shows promising results that require further studies to validate their robustness and reliability through large-scale trials introduced in clinical practice. Even though liquid biopsy cannot wholly replace EMB, these two diagnostic approaches can be combined in clinical practice.

The synergic power of these two approaches can increase the accuracy of cardiac allograft rejection diagnosis. Defining the most effective rejection monitoring strategy could be the driving force for the settlement of a multimodal approach toward HT patient management.

The dynamic landscape of rejection surveillance, described in this review, highlights the evolution of the concept of EMB from a necessary procedure for histopathological evaluation of the transplanted heart state towards a comprehensive molecular resource accompanied by liquid biopsy. This holistic view of the follow-up patient’s pathway brings to light a consistent multimodal personalized approach with the direct integration in clinical practice of invasive and non-invasive procedures, leading to a progressive change of paradigm in heart transplant monitoring.

## Figures and Tables

**Table 1:** Emerging biomarkers in the diagnosis of allograft rejection after heart transplantation.

Study	Diagnostic Tool	*n*. of patients	Area Under the Curve	Sensitivity (%)	Specificity (%)
Pham MX. 2010 [7]	GEP of peripheral specimen (randomized controlled trial: Image study)	602	Not inferior to EMB		
Deng MC. 2006 [8]	GEP of peripheral blood leucocytes (11-gene real time PCR: Cargo study)	170	0.686–0.914	75.8 *	41.8 *
Di Francesco A. 2018 [10]	Tissue microRNAs (combination of miR-208a-5p, -126-5p, -135-5p)	33	0.951–1.000	83.3	95.8
Nováková T. 2019 [11]	Tissue microRNAs (combination of miR-144, 589, 146, 182, 3135b, 10, 31, 17, 1273, 3605, 4506)	38	0.72–0.96	68	91
Kobashigawa J. 2015 [16]	GEP of peripheral specimen (randomized controlled trial)	60	Not inferior to EMB		
Crespo-Leiro MG. 2016 [17]	GEP of peripheral specimen (observational study: Cargo II study)	462	0.690–0.700	86.4 *	46.5 *
Shannon CP, 2020 [19]	Whole blood transcriptome profile (nine mRNA transcript Nanostring nCounter)	177	0.69 | 0.70	89	47
Lin D. 2009 [21]	Whole blood genomic profile (12-gene biomarker panel)	28	N.A.	83.0	100.0
Hollander Z. 2010 [22]	Whole blood genomic profile (Affymetrix Human Genome U133 Plus 2.0 chips)	31	0.600–0.830	N.A.	N.A.
De Vlaminck I. 2014 [26]	Quantification of circulating cell-free donor-derived DNA	65	0.830	58.0	93
Khush KK. 2019 [30]	Quantification of circulating cell-free donor-derived DNA	676	0.64	44.0	80
Richmond ME. 2020 [31]	Quantification of donor fraction of cell-free DNA (0R vs. ≥ 1R)	174	0.86	80	88
Sukma Dewi I. 2013 [45]	Identification of single serum microRNA (miR-326, miR-142-3p)	10	0.800–0.860	N.A.	N.A.
Sukma Dewi I. 2017 [46]	Identification of single serum microRNA (miR-101-3p, miR-142-3p)	63	0.750–0.780	N.A.	N.A.
Duong VH JP. 2014 [47]	Identification of single serum microRNA (miR-10a, -31, -92a, -155)	113	0.867–981 *	N.A.	N.A.
Castellani C. 2020 [58]	Characterization of circulating extracellular vesicles surface antigens	90	0.727−0.939	100.0 *	85.7 *
Kennel PJ. 2018 [59]	Serum exosomal protein profiling	48	N.A.	N.A.	N.A.
Halloran PF. 2017 [72]	Microarray-based molecular microscope (MMDx System)	221	0.650–0.810	N.A.	N.A.
Bodez D. 2016 [76]	GEP of myocardial tissue (combination of 15 genes—the GET study)	30	N.A.	100.0	100.0
Loupy A. 2017 [77]	GEP of myocardial tissue (four different gene sets)	110	0.800–0.870 *	N.A.	N.A.
Afzali B. 2017 [78]	GEP of myocardial tissue (three different gene sets)	163	0.778 *	46.5 *	80.0 *

* Performance at validation. N.A., not assessed; GEP, gene expression profiling.

## Data Availability

No new data were created or analyzed in this study. Data sharing is not applicable to this article.
